# Geological and Pleistocene glaciations explain the demography and disjunct distribution of red panda (*A. fulgens*) in eastern Himalayas

**DOI:** 10.1038/s41598-020-80586-6

**Published:** 2021-01-08

**Authors:** Supriyo Dalui, Sujeet Kumar Singh, Bheem Dutt Joshi, Avijit Ghosh, Shambadeb Basu, Hiren Khatri, Lalit Kumar Sharma, Kailash Chandra, Mukesh Thakur

**Affiliations:** 1 Zoological Survey of India, New Alipore, Kolkata, West Bengal 700053 India; 2 Department of Zoology, University of Calcutta, Kolkata, West Bengal 700019 India

**Keywords:** Evolution, Genetics, Zoology, Ecology, Animal migration, Biodiversity, Conservation biology, Ecological genetics, Molecular ecology

## Abstract

Pleistocene glaciations facilitated climatic oscillations that caused for enormous heterogeneity in landscapes, and consequently affected demography and distribution patterns of the mountain endemic species. In this context, we investigated demographic history and population genetic structure of red panda, distributed along the geographical proximity in the southern edge of the Qinghai-Tibetan Plateau. Bayesian based phylogeny demonstrated that red panda diverged about 0.30 million years ago (CI 0.23–0.39) into two phylogenetic (sub) species, that correspond to the middle-late Pleistocene transition. The observed intraspecific clades with respect to Himalayan and Chinese red panda indicated restricted gene flow resulting from the Pleistocene glaciations in the eastern and southern Tibetan Plateau. We found Himalayan red panda population at least in KL-India declined abruptly in last 5–10 thousand years after being under demographic equilibrium. We suggest revisiting the ongoing conservation activities through cross border collaboration by developing multi-nationals, and multi-lateral species-oriented conservation action plans to support the red panda populations in transboundary landscapes.

## Introduction

Pleistocene climatic oscillations driven circadian cooling and warming in the landscapes caused periodic range fluctuations and facilitated to shape distribution, population sizes and demography of the mountain dwelling species in east Asia^1,2^. More specifically, Himalayan region experienced four major Pleistocene glaciations events^3–5^, that formed varying paleo-ecological niches^6^, and profoundly impacted on the distribution patterns of several Himalayan endemic species^2,4,7,8^. In addition, complex and multifaceted topography of the mountain ranges affected the Himalayan endemic species by facilitating events like colonization^9,10^ or formation of small and isolated patches in the landscape^11,12^. A few studies from Himalayan region and Tibetan plateau already exhibited changes in the species demography in response to the historic events^13–15^. In particular to the glaciation events, ecologically specialists and those species that have small home ranges, have been affected more than the widespread and ecologically generalist species^16–18^. Therefore, to understand the impact of past climatic oscillations on an ecologically specialist species like red panda, it would be imperative to make reasonable predictions of the climate change effects on the existing species that share habitat, resources or sympatric in their ecological needs.

Red panda (*Ailurus fulgens*), a magnificent iconic species of Central and Eastern Himalayas, has lost its potential habitat and declined abruptly over the last 20 years by losing about 50% of its wild population across the range with a probable 2500 viable individuals left in the wild^19^. Presently, red panda is categorized as *‘Endangered*’ under the IUCN Red list^19^ and protected as *Schedule I* in the Wildlife (Protection) Act, 1972 of India^20^. Historically, the red panda was distributed across Eurasia but due to the adverse conditions, its present distribution restricted only to the temperate conifer and cool broadleaf forest of Nepal, India, Bhutan, Myanmar, and southwest China^21^. Nepal, India and Bhutan fall within the Himalayan range, while the easternmost distribution in China (Hengduan Mountain) is beyond the Himalayan range. Based on the morphological characters, red panda was taxonomically classified into two subspecies, *Ailurus fulgens fulgens,* reported to be distributed in Nepal, India, Bhutan, Myanmar, and China (Tibet and western Yunnan province) and *Ailurus fulgens styani*, distributed in the Sichuan and Yunnan provinces of China^22^ and Nujiang river was believed to be a biogeographic barrier for the separation of the two subspecies^23^. However, earlier studies that evaluated evolutionary history of red panda, did not observe any significant genetic differentiation between the two subspecies^24–26^. Recently, Hu et al. demonstrated the presence of two phylogenetic species, the Himalayan red panda (*Ailurus fulgens*; henceforth HRP) and Chinese red panda (*Ailurus styani*; henceforth CRP), and proposed that Yalu Zangbu river has been the potential boundary of species divergence^27^. This study sequenced 18 HRPs from Nepal, and inferred that it suffered from three historic bottlenecks followed by a small expansion consequently imparting low genetic diversity. Further, earlier genetic studies on CRP explained that red panda populations maintained genetic variability^24–27^. Additionally, the historical events impacted the species demography and this can be well observed in the species distribution patterns^21,27,28^. In China, red panda populations have experienced large-scale habitat loss and fragmentation^26,27^. While, red panda populations in Kangchenjunga landscape (KL)-India were recently examined and observed to exist in meta-population frame work, which reflected the functionality of the connecting corridors between the habitat patches and support the viable populations of red panda^28^. In the present study, we aimed to explore the phylogeographic patterns and demographic history of red panda from Indian Himalayan Region (IHR) in the context of the historic Pleistocene climatic glaciations.

## Results

### Phylogenetic analysis

**Figure 1:**
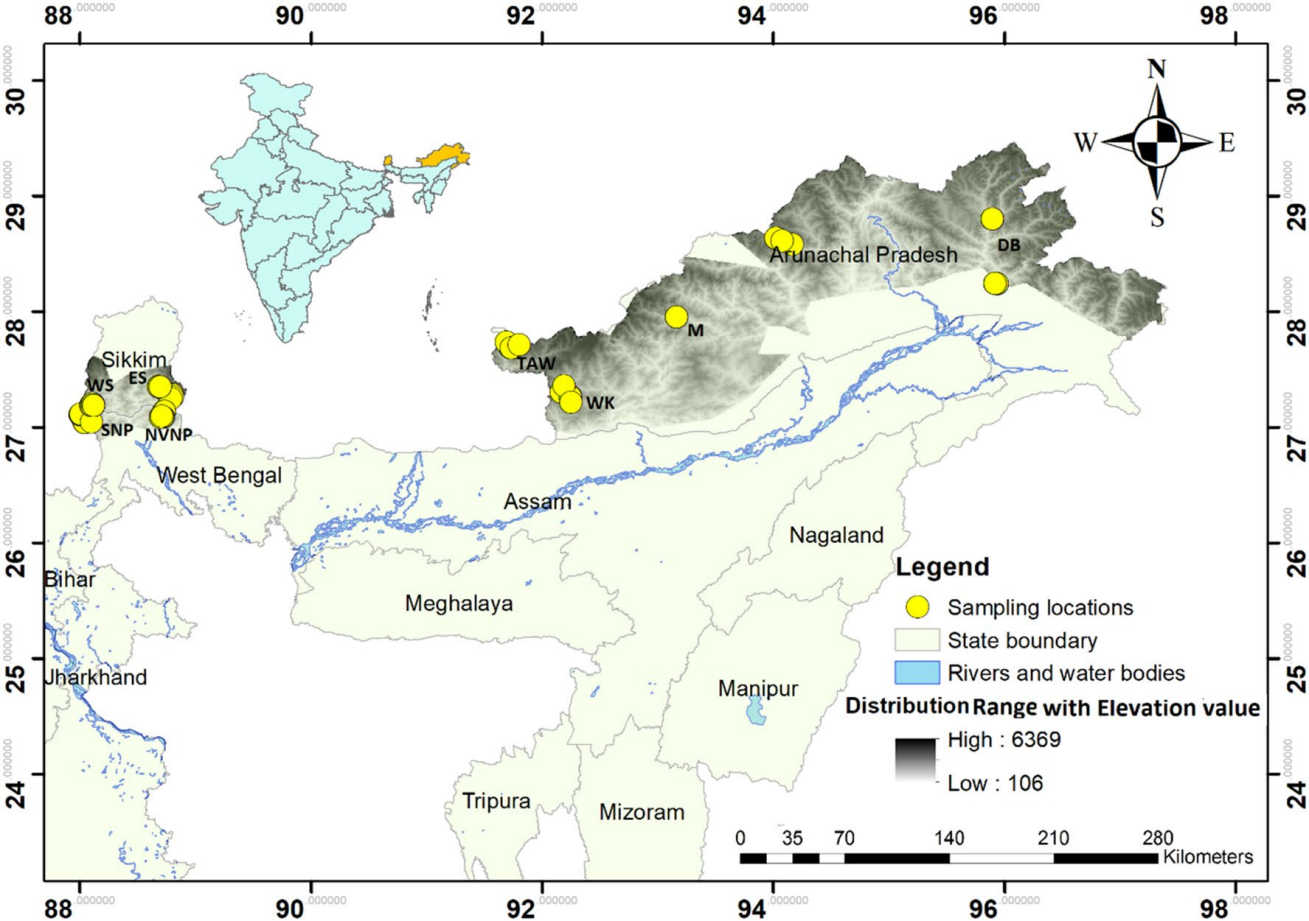
Study area map showing the sampling locations in Central and Eastern Himalayas of India. The coloured gradient represents the IUCN Red panda distribution range in India with elevation range. The abbreviations which denote the areas of the haplotypes are as follows. *SNP* Singhalila National Park, *WS* west Sikkim, *ES* east Sikkim, *NVNP* Neora valley National Park, *TAW* Tawang, *WK* west Kameng *M* Menchuka, *DB* Dibang Valley. (Map generated using ArcGIS: https://www.arcgis.com/index.html).

**Figure 2:**
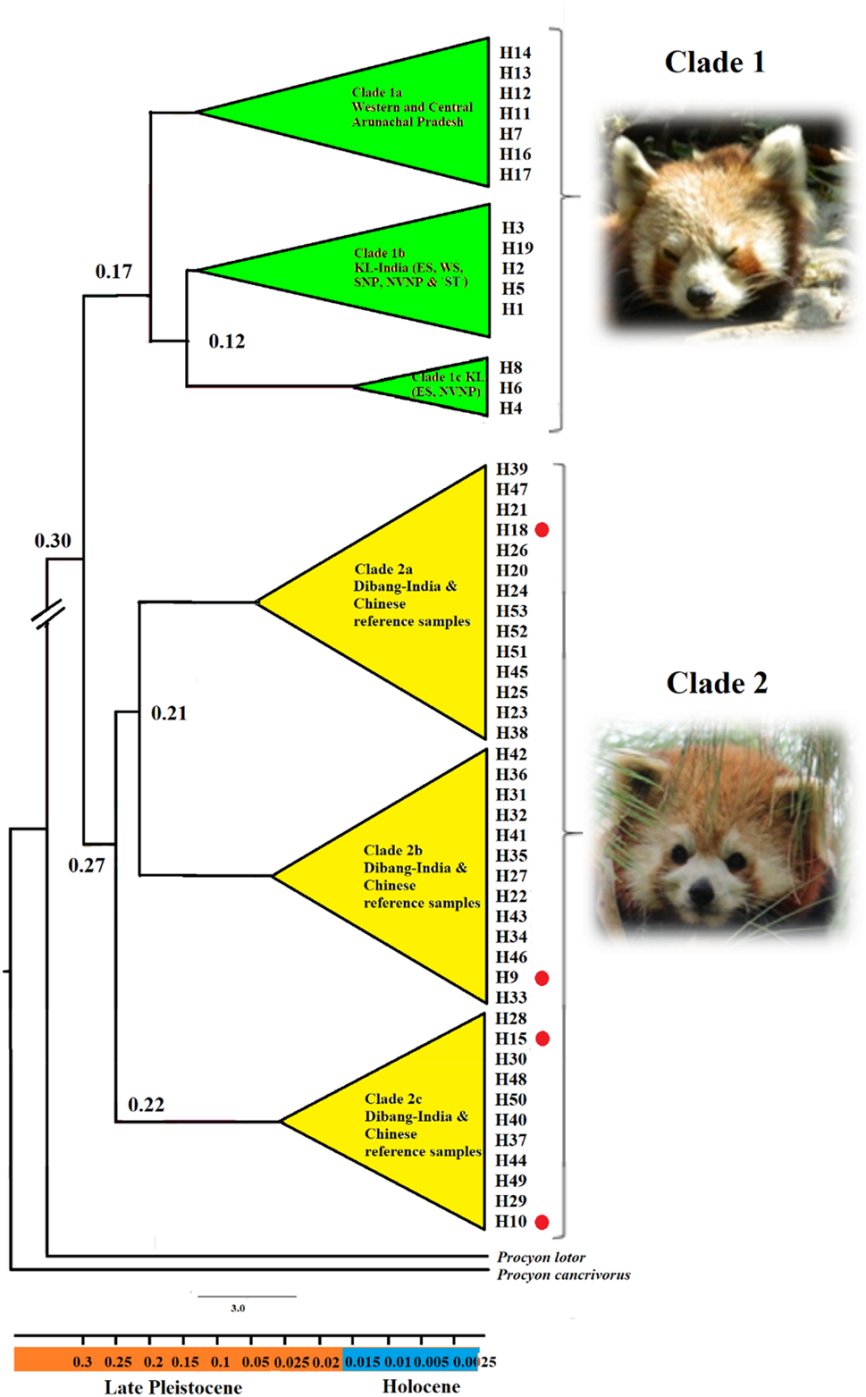
Re-construction of Bayesian based phylogenetic relationship using mitochondrial control region **(**Mentioned values on the nodes indicated the divergence time estimated in million years ago (median divergence estimate). Clade 1a represented western and central Arunachal, 1b represented KL-India and Tibet, and 1c represented clade KL-India, clade 2a, 2b, 2c represented the haplotypes from Dibang valley (H9, H10, H15, H18 marked with red circles) and haplotypes of the sequences of Chinese red panda retrieved from NCBI GenBank.

We successfully extracted DNA from 90 scats out of 132 scat processed and a total 44 samples (48%) yielded high quality control region sequences (Fig. 1). Genetic analyses of 44 sequences of IHR yielded 18 unique haplotypes characterized by 34 polymorphic sites (Supplementary Table S1). We also mined 44 sequences of CRP available on public domain (Supplementary Table S2). Bayesian based phylogenetic analysis grouped all the sequences into two major clades—clade1 and 2 in accordance to the HRP and CRP, respectively (Fig. 2). The sub-clade 1a in the major clade 1, represented sequences originated from West and Central Arunachal Pradesh while clade 1b and 1c contained sequences originated from KL-India (SNP, WS, ES and NVNP) along with two sequences retrieved from south Tibet (ST) (GenBank HQ992985 and AY849727). The clade 2 comprised of the sequences originated from the mountains of southwest China and DB in the east Arunachal Pradesh. Further, the HRP and CRP clades were found to be diverged about 0.30 mya (95% CI 0.23–0.39), that correspond to the middle-late Pleistocene transition (Fig. 2). Whereas, clade 1a of west and central Arunachal Pradesh diverged from clade 1b (KL and south Tibet) and clade 1c (KL) 0.17 mya. The clade 1c of KL population evolved individually about 0.12 mya after separation from clade 1b. Red panda population of DB diverged from CRP and found relatively old than the KL and west and central Arunachal Pradesh population, indicating DB population as an ancestral population from which HRP diverged.

### Phylogeographic and population genetic structure

**Figure 3:**
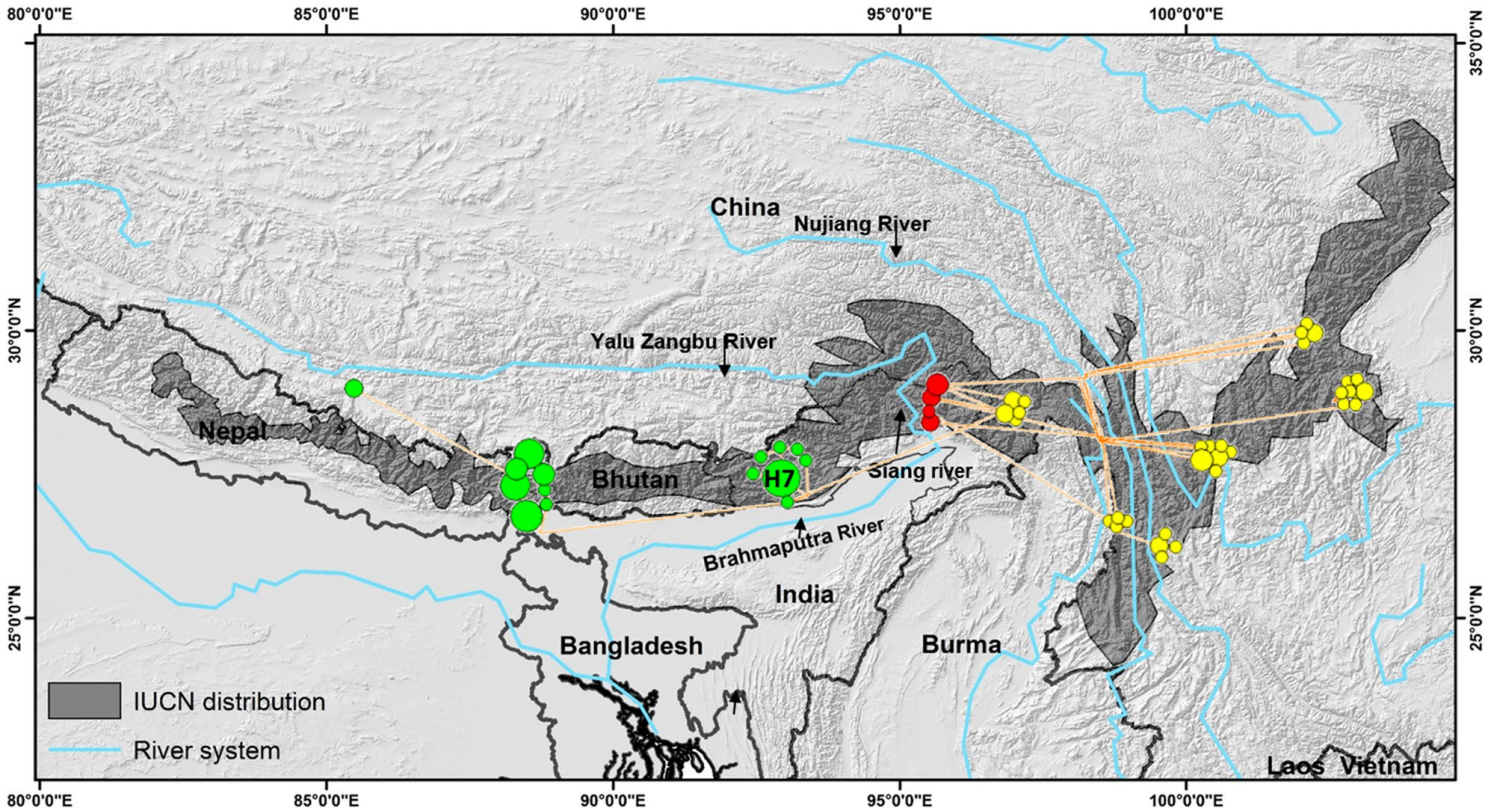
Distribution of haplotype with respect to the geographical origin of the samples in India and China. Green color dots represented Himalayan red panda, Yellow dots represented Chinese red panda (samples originated from the administrative boundary of China and red color represented Chinese red panda observed in the Dibang valley, Arunachal Pradesh, India). (Map generated using ArcGIS: https://www.arcgis.com/index.html).

We obtained total 53 haplotypes 15 unique to HRP and 38 unique to the CRP from the samples collected from IHR and sequences obtained from public domain (Table 1). Further, in coherence to the phylogenetic analysis, network also segregated haplotypes in accordance to the HRP and CRP clades (Fig. 3, Table 1). Haplotypes (H1–H6 and H8) originated from KL, TAW (H7), WK (H7, H11–H14, H17), M (H16) and ST (H19) were clustered in one major group belonged to HRP while haplotypes originated from DB (H9, H10, H15 and H18) and China H20–H53) clustered with the second major group belonged to CRP (Figs. 2 and 3, Table 1). H1–H6, H8 and H19 correspond to the haplotypes of Clade 1b and 1c, representing population of KL-India i.e. SNP, ES, WS and NVNP and ST (Fig. 3; Table 1).

**Table 1:** Observed haplotypes in red panda across its range.

Geographical regions	No. of sequences used	Species	H1	H2	H3	H4	H5	H6	H7	H8	H9	H10	H11	H12	H13	H14	H15	H16	H17	H18	H19	H20 to H53
SNP	2	*A. fulgens*	0	2	0	0	0	0	0	0	0	0	0	0	0	0	0	0	0	0	0	0
WS	5	*A. fulgens*	5	0	0	0	0	0	0	0	0	0	0	0	0	0	0	0	0	0	0	0
ES	10	*A. fulgens*	0	1	6	0	1	0	0	2	0	0	0	0	0	0	0	0	0	0	0	0
NVNP	7	*A. fulgens*	0	2	0	1	2	1	0	1	0	0	0	0	0	0	0	0	0	0	0	0
TAW	4	*A. fulgens*	0	0	0	0	0	0	4	0	0	0	0	0	0	0	0	0	0	0	0	0
WK	9	*A. fulgens*	0	0	0	0	0	0	4	0	0	0	1	1	1	1	0	0	1	0	0	0
M	1	*A. fulgens*	0	0	0	0	0	0	0	0	0	0	0	0	0	0	0	1	0	0	0	0
ST	2	*A. fulgens*	0	0	0	0	0	0	0	0	0	0	0	0	0	0	0	0	0	0	2	0
DB	6	*A. styani*	0	0	0	0	0	0	0	0	2	2	0	0	0	0	1	0	0	1	0	0
CHINA	42	*A. styani*	0	0	0	0	0	0	0	0	0	0	0	0	0	0	0	0	0	0	0	42

*SNP* Singhalila NP, *WS* west Sikkim, *ES* east Sikkim, *NVNP* Neora valley NP, *TAW* Tawang, *WK* west Kameng, *M* Menchuka, *DB* Dibang valley, *ST* south Tibet, China.

**Figure 4:**
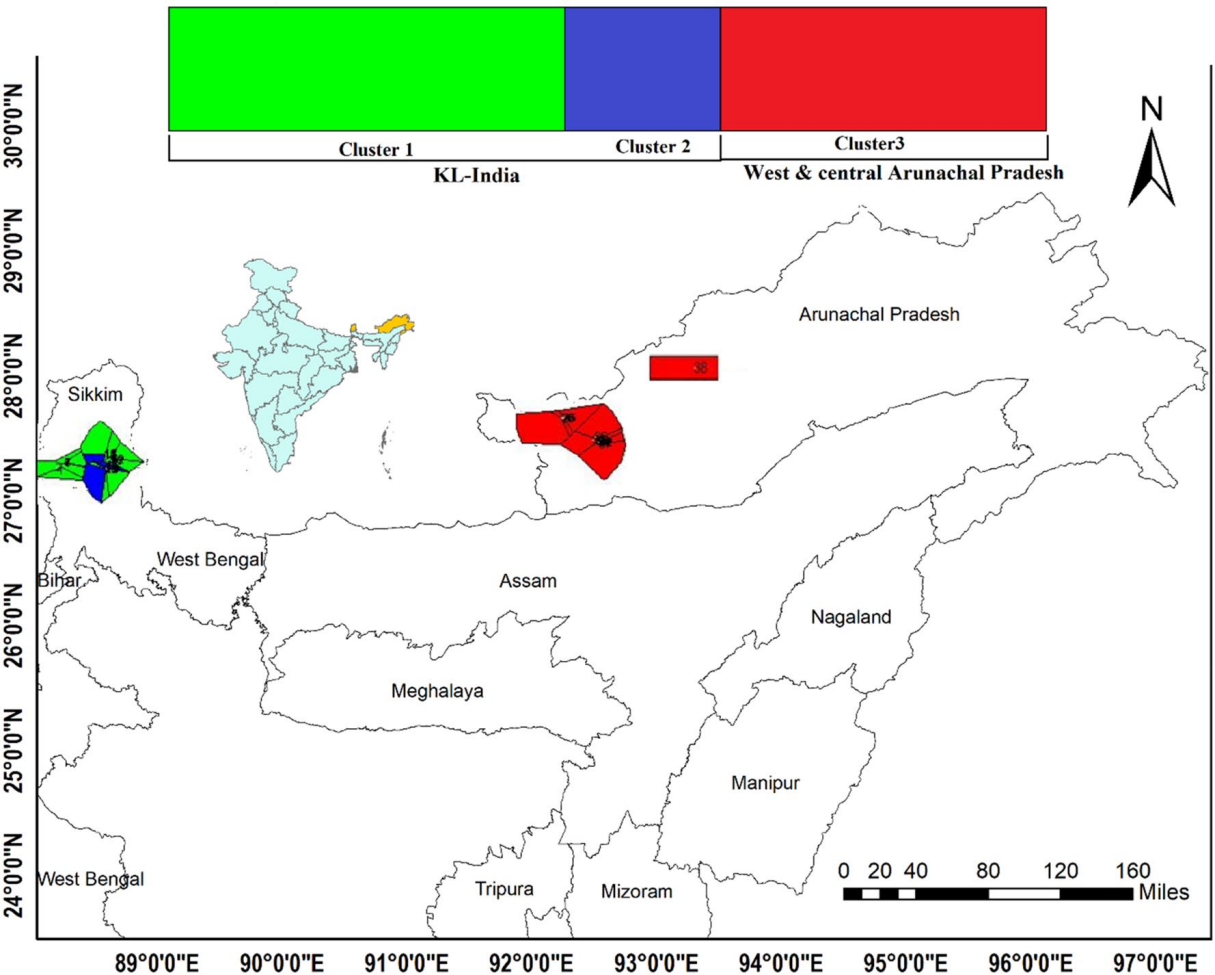
Bayesian analysis of population structure (BAPS) of *A. fulgens* interpreted three population cluster. Cluster 1 and 2 in green and blue colour representing KL-India population and cluster 3 in red colour representing west and central Arunachal Pradesh population of HRP. The Voronoi polygons on the map also represented the population clusters. (Map generated using ArcGIS: https://www.arcgis.com/index.html.

In HRP, we obtained 15 unique haplotypes characterized by the 25 polymorphic sites with an average nucleotide difference of 5.926 (Table 2). The overall *Hd* and *π* were 0.910 (± 0.022) and 0.014 (± 0.0013). While in CRP, we obtained 38 haplotypes characterized by 41 polymorphic sites with an average nucleotide difference of 8.716. The overall *Hd* and *π* were 0.989 (± 0.007) and 0.019 (± 0.0009) (Table 2). The haplotype H7 formed the base of the other haplotypes i.e. H11–H14, H16–H17 originated from west and central Arunachal Pradesh indicating other haplotypes descended from the haplotype H7 (Fig. 3). BAPS also indicated the presence of three populations of HRP, two in KL and one in the west and central Arunachal Pradesh (Fig. 4). The results of SAMOVA revealed three groups in red panda with 46.23% variation among groups and 4.38% among populations within groups and 49.40% variation within populations. The significant *F*ST showed a high genetic differentiation and low gene flow between populations (Table S3, Fig. S1).

**Table 2:** Summary of genetic diversity indices and neutrality tests of demographic expansion of red panda populations.

Diversity estimates						Neutrality tests				Mismatch distribution	
N	P	H	K	Hd	π	Tajima’s D	Fu's Fs	Fu and Li’s D	Fu and Li’s F	SSD	Rg
**Himalayan red panda (A. fulgens)**											
40	25	15	5.926 ± 2.89	0.910 ± 0.022	0.014 ± 0.0013	0.0278 *P* = 0.49	− 1.213 *P* = 0.36	0.16814 *P* = 0.55	0.10504 *P* = 0.55	0.024 *P* = 0.07	0.049 *P* = 0.06
**Chinese red panda (A. styani)**											
48	41	38	8.716 ± 4.094	0.989 ± 0.007	0.019 ± 0.0009	− 0.194 *P* = 0.43	− 24.199 *P* = 0.00	− 0.165 *P* = 0.403	− 0.235 *P* = 0.38	0.00127 *P* = 0.64	0.0059 *P* = 0.70

N—Number of samples; P—Polymorphic sites; H—Number of Haplotypes; K—Average number of nucleotide differences; Hd—Haplotypes diversity; π—Nucleotide diversity; Neutrality tests with *P* > 0.5; SSD—Sums of squared deviations; Rg—Harpending’ sraggedness index.

### Demographic history

**Figure 5:**
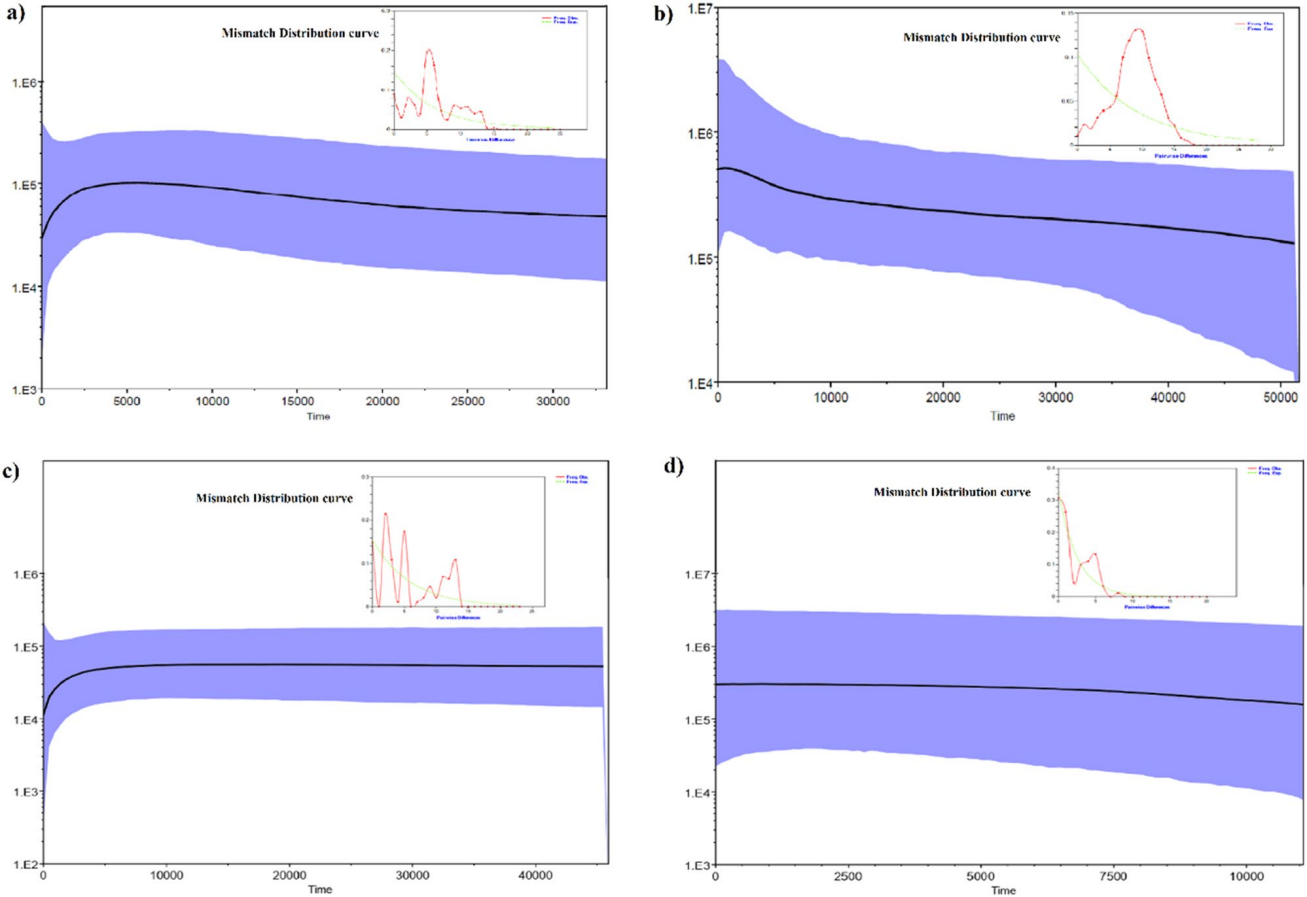
Demographic history of red panda populations estimated using Bayesian skyline plot (BSP). (**a**) Himalayan red panda, (b) Chinese red panda, (c) Himalayan red panda- KL India population and (**d**) Himalayan red panda- Arunachal Pradesh population. The solid line shows the median estimates of Ne τ (Ne = effective population size; τ = generation time), and the blue area around median estimates show the 95% highest posterior density (HPD) estimate of the historic effective population size. The timing of events was estimated assuming a substitution rate of 1.2 × 10^–7^ calculated for red panda. Mismatch distributions curves of pairwise differences of each red panda populations are shown within the corresponding BSP graph.

For HRP, Tajima’s D was positive and significant (0.0278, *P* = 0.49), that suggested population under equilibrium while Fu’s Fs statistic tests was negative and suggested population under expansion (− 1.213; *P* = 0.36; Table 2). Fu and Li’s D and F tests also indicated no significant departure from neutrality (*P* > 0.5). Further, we obtained a multimodal and ragged shaped mismatch distribution curve (Fig. 5a), indicating population under demographic equilibrium and did not undergo any population expansion. The mismatch distribution curve did not fit for sudden expansion in goodness-of-fit test (SSD 0.024, *P* = 0.07). Further, the multimodal mismatch distribution with a high non-significant raggedness index (*Rg* 0.049 *P* = 0.06), suggested that population had maintained relatively long-term demographic stability. Neutrality tests in general suggested a long-term historical demographic stability in HRP population except Fu’s Fs statistics. Coalescent based BSP showed that HRP (by pooling all sequences) was under a long demographic equilibrium but then experienced a recent sharp decline in the effective population size over the last 5 to 10 ky (Fig. 5a). Interestingly, while analyzing populations of HRP, no significant difference in the changes of the effective population size were observed in case of KL-India population. However, the HRP population in Arunachal Pradesh seemed to be under demographic equilibrium (Fig. 5c,d).

In contrary, neutrality tests showed CRP had experienced population expansion in the past (Tajima’s D = − 0.194, *P* = *0.43*, Fu’s Fs = − 24.19, *P* = 0.00 and Fu’s D = − 0.165; *P* = 0.403). Further, unimodal mismatch distribution curve, a non-significant raggedness (0.0059; *P* = 0.07) as well as SSD (0.00127; *P* = 0.64) also supported historical population expansion in CRP (Table 2; Fig. 5d). The observed pattern of BSP supported that CRP remained demographically stable and experienced a population expansion in the last 5 to 10 k (Fig. 5b).

## Discussion

The observed clustering patterns were in coherence to the recent phylogenetic species classification of red panda^27^. However, we propose '*Siang River*' that flows in the downstream to Yalu Zangbu River is the potential barrier for species divergence in red panda. It is noteworthy to mention that Yalu Zangbu and Siang rivers are essentially the same river flow at different stretches in Tibet and India. However, Yalu Zangbu originates at Angsi Glacier in the western Tibet, southeast of Mount Kailash and Lake Mansarovar vary in topographic features like raggedness, elevation, depth, breath, current flow, seasonality, etc. at different region (from Tibet to Arunachal Pradesh to Assam)^29–31^. Further, Yalu Zangbu river descends from 4500 to 3000 m and surrounding vegetation changes from cold desert to arid steppe to deciduous scrub vegetation and this further changes into a conifer and rhododendron forest when enter to Arunachal Pradesh, India^32^. Thus, changes in the mentioned topographic features and habitat types often influence the distribution and movement of animals across the river. Hence, it is imperative to mention the Siang River, the regional stretch of Yalu Zangbu River in western Arunachal Pradesh, India is responsible to hinder the movement of red panda across the range. We observed that samples collected from DB in the east of Siang River clustered with the CRP and two sequences retrieved from Li et al.^25^ originated from South Tibet, were clustered with HRP (Fig. 3). A few studies reported that large rivers often function as barrier in the distribution and gene flow of the arboreal and small mammals^33–35^. Thus, occurrence of *A. styani* in DB that lies on the east of Siang river was in accordance to the fact that Siang river is a potential geographic barrier in several species including Hoolock Gibbon, Stump tailed macaque, Pigtailed macaque^33^.

### Phylogeographic structure

We observed adequate genetic variation in HRP and CRP and the observed diversity estimates were comparable with the earlier studies on red panda from China^24–26^. Further, Hu et al. reported that HRP had experienced three historical bottlenecks and showed low nuclear genetic diversity based on the samples collected from Nepal^27^. In contrast, we did not observe a low genetic variation in control region of mtDNA. Such type of the disagreement between nuclear and mtDNA genetic diversity has been observed in many groups of taxa because of the differential relationship of population size and genetic variation. Study detected no correlation between variation in mtDNA and population size in large body mammals while nuclear data genetic variation is fairly consistent with population size^36^. Although, we observed a relatively high genetic variation in HRP based on partial fragment of control region, but the existing anthropogenic threats are so grave that wild populations of HRP declined abruptly over the last 20 years by losing 50% population across the range^19^.

Among observed clusters of HRP and CRP, the phylogenetic tree revealed the presence of three subclades (1a, 1b, 1c) in HRP (Figs. 2 and 3). The formation of two clades in KL, particularly 1c present in the ES (haplotypes H4, H6 and H8) possibly indicated a cryptic population signature of the adjacent populations of Bhutan. Interestingly, one haplotype from ST (South Tibet), clustered in clade 1b with haplotypes from SNP, ES and WS, clearly indicated the distribution of HRP population from west of the Siang river to Tibet including distribution in Nepal and Bhutan. Further, BAPS analysis also indicated the presence of three clusters, and revealed the presence of geographic structure, where two populations / sub-populations were observed in KL- India and one in Arunachal Pradesh (Fig. 4). Similarly, SAMOVA results indicated three groups (Fig. S1) in accordance to the geographical distribution of the clades. High level of FST (0.506) among the groups indicated strong genetic structure based on the geographic distance and barriers (Table S3). The observing two isolated populations of HRP in KL-India was also in accordance to our previous study that supported that red panda exist in meta-population framework in KL-India^28^.

### Population demographic history and divergence

The estimates of neutrality tests of HRP (Tajima’s D, Fu’s and Fu’s D) were not consistent. However, multimodal mismatch distribution curve (Fig. 5a) indicated population under demographic equilibrium and did not experience a population expansion in the past. In contrary, BSP demonstrated an apparent evidence of recent reduction in the effective population size in last 5 to 10 kya within the middle to late Holocene. (Fig. 5a). This disagreement plausibly observed due to retention of the structured HRP population while analyzing the changes in the effective population size as BSP like many other coalescent-based methods infer the demographic history of populations assuming a single, isolated and panmictic population^37^. However, population wise assessment of HRP indicated that populations in KL-India and in Arunachal Pradesh would have experienced varying threats or historic events in the past that imparted differences in the changes in the effective population sizes over time (Fig. 5c,d). The KL-India HRP population appeared to experience a recent decline in the population size and may be attributed to the factors emerged in last 5–10 kya within the middle to late Holocene. Further, given the spatial position and having transboundary distribution, HRP population decline may be attributed to the miscellaneous effects of various anthropogenic activities emerged in last few decades and also the middle to late Holocene climate fluctuations. In contrary, *A. styani* was found under expansion and demographically stable over the last 5–10 ky (Fig. 5b). The species divergence history showed an initial split occurred about 0.30 mya between the HRP and CRP and thereafter, both species evolved independently. The DB population, being in the eastern edge of Siang river evolved with the CRP and further diverged into three different lineages (clade 2a, 2b,2c). While in HRP, the clade 1b and 1c of KL and 1a clade of west and central Arunachal population diverged about 0.17 mya during the Penultimate glaciation and interestingly the individuals of clade 1c of KL population emerged independently about 0.12 mya.

Further, the upliftment of the Tibetan Plateau greatly affected the geological environment by forming several new mountain ranges and has been hypothesized to induce phylogeographic splitting within and or among species in the southeast Asia, including species both on and outside of the plateau e.g. Giant panda^38^, Asian great tit^14^. Major geographic and environmental transitions occurred during 55–15 mya^39–41^ or earlier^42^. In Bayesian analysis, red panda species divergence is estimated to occur in the middle-late Pleistocene transition i.e. 0.30 mya (0.23–0.39). Further, the comparative phylogeographic studies in the Tibetan plateau and Himalayan ranges have shown that species distributed mainly in the heavily ice-covered regions experienced population expansion afterward the glaciation events, whereas the species distributed on the ice-free edges of the plateau maintained their population size at a stable level^13,38^. Demographic stresses on the edge species is endured as they confronted milder climate in comparison to their platform-distributed counterparts. Multiple fluctuations of cooling and warming events during the Pleistocene transition, probably contributed mixed effects of population contraction and expansion events. Further, the different clades did not observe to diverge at same rate due to the varying geographical complexity and environmental heterogeneity. Therefore, we hypothesize that Pleistocene climate-driven vicariant events would have been responsible for the observed demography and divergence patterns in red panda. The CRP as distributed chiefly in the Hengduan mountains, geographically away from the edges of the Qinghai-Tibetan Plateau and, possibly less suffered from the impact of the Pleistocene glaciation transitions^23^. In contrary, HRP experienced the severity of the Pleistocene glaciation transition and the interglacial period since HRP occurred in the southern edge of the Qinghai-Tibetan Plateau with geographic proximity to glaciers. The substantial loses of the suitable habitat of HRP during the ice age might have resulted in slow recovery of this precarious species. Further, in the recent past, the emerging human activities and changes in the land use patterns also impacted red panda populations more than the climate change effects^27^.

## Conclusion

We demonstrated that IHR harbours the habitat and occurrence of both the phylogenetic species of red panda, which diverged by Siang river in Arunachal Pradesh. The clear understanding of distribution boundary between two species will halt the inappropriate conservation action and avoid detrimental interbreeding between the two species in captivity^43,44^. Further, the presence of temporal subclades in HRP reflected the ecological responses to the climatic fluctuations in Pleistocene and increasing human activities in the recent past. The present study will help to gain insights into the effects of geological events on the population divergence and distribution of allele frequencies in the landscape which provide vital information for making species recovery programs^45,46^. This study has been the first exhaustive attempt to investigate red panda demography from IHR. Being a habitat specialist and cold tolerant Himalayan endemic species, red panda is more vulnerable to the recent fluctuation in temperature and anthropogenic activities. In particular, the conservation status and population size of DB of CRP and KL population of HRP require further evaluation. The HRP population in KL needs special attention as it represents an intermediate population that connects to Nepal in the west and Bhutan in the east. The existence of red panda in meta-populations frame work in KL^28^, provide opportunities to physically connect the habitat patches so that forest patches can be functionally operated by red panda and avoid the local extirpation of small populations. We propose to develop a strong transboundary collaboration to entail a cross-border monitoring network by involving active contribution of bureaucrats, local stakeholders, environmentalists, biologists, NGOs and communities among India, Nepal and Bhutan.

## Materials and methods

### Study area and sample collection

We received necessary permissions for undertaking field survey and collection of faecal samples from the State forest departments of West Bengal (F.No. 1689/WL/2M-126/2018), Sikkim (F.No. 78/GOS/FEWMD/BDR/PCCF-Secy/CWLW85) and Arunachal Pradesh (F.No. CWL/Gen/173/2018-19/Pt. VIII/ 1518-24). Our sampling efforts covered protected areas (PAs) like- Singhalila National Park (SNP) and Neora valley National Park (NVNP) in the Northern West Bengal, about 10 PAs in the West Sikkim (WS) and East Sikkim (ES), along with Tawang (TAW) and West Kameng (WK) district in the western Arunachal Pradesh, Menchuka (M) from central Arunachal Pradesh and Dibang valley (DB) district in the eastern Arunachal Pradesh. In total, we collected 132 faecal samples- 57 samples from KL in the Central Himalayas (29 from North West Bengal and 28 from Sikkim) and 75 samples from Arunachal Pradesh in the Eastern Himalayas (Fig. 1). We stored samples in 70% ethanol immediately after collection from the field and transported to laboratory for further processing. We kept samples at − 20 °C until processing for DNA extraction.

### PCR amplification and sequencing

We extracted genomic DNA using QIAamp Fast DNA Stool Mini Kit (QIAGEN Germany) following manufacturer's instructions and sequenced a 440 bp control region fragment of mitochondrial DNA using red panda specific primers following Su et al., and Li et al.^24,25^. PCR amplifications were performed on Veriti thermal cycler (Applied Biosystems, USA) with a total volume of 10 μl comprising of 20–30 ng of template DNA, 1U Taq polymerase (Takara), 10× PCR buffer, 1 mM MgCl_2_, 2.5 mM dNTPs mix, 0.1 µM of each primer, 0.1 µg/µL BSA. Thermal cycling conditions were as follows: an initial denaturation at 94 °C for 5 min, followed by 35 cycles at 94 °C for 30 s, 50 °C for 45 s and 72 °C for 1 min. The final extension was at 72 °C for 10 min. Sequencing was performed using Big-Dye Terminator Cycle Sequencing Kit 3.1 (Thermo Scientific, USA) on an ABI 3730 Genetic analyzer (Applied Biosystems, USA). We also retrieved 44 sequences of control region of red panda available in the public domain (Supplementary Table S2).

### Phylogenetic analysis

Genetic diversity estimates i.e. haplotype diversity (*Hd*), number of polymorphic sites (*P*), mean number of pairwise nucleotide differences (k) and nucleotide diversity (*π*), were estimated using DnaSP 6^47^. To infer the evolutionary relationships, we performed Bayesian based phylogenetic analysis in BEAST 2^48^. The Hasegawa-Kishino-Yano (HKY) mutation model was selected as the best nucleotide substitution model for the analysis based on the Bayesian information criterion (BIC) using MrModeltest v2.3^49^. We used a strict molecular clock model to yield effective sample sizes (ESS) taking a substitution rate of 1.2 × 10^–7^ calculated for red panda. The substitution was calculated using formula µ = D × g/2t taking procyonids as comparative species. Where the D was pairwise difference between the two species (1.2), g was generation time (6 year) and T was the estimated divergence time between two species (29.5; Sato et al.)^50^. We considered raccoon (*Procyon lotor* and *Procyon cancrivorus*) sequences as outgroup and the phylogenetic analyses were conducted for 2 × 10^8^ generations each with sampling every 10,000 generations. We checked the performance using Tracer1.5^51^ and accepted the results if ESS > 200. The consensus tree was generated using Tree Annotator, after a burn-in of 25% and visualized in FigTree1.4.3^52^. We considered groups which were supported by a posterior probability value > 95%.

### Phylogeographic structure

The median-joining network calculations were carried out by assigning equal weights to all variable sites and with default values for the epsilon parameter (epsilon = 0) to minimize alternative median networks using NETWORK 4.5.1.0 (www.fluxus-engineering.com)^53^. Further, we inferred the population structure using BAPS 6 with prior information of the sampling location assuming spatial admixture model^54,55^. We also conducted non-Bayesian cluster analysis using SAMOVA 2.0 using sampling locations. Analyses were performed following 100 simulated annealing steps with increasing number of groups (K) from 1 to 6^56^.

### Demographic history

Demographic parameters like mismatch distribution test (expansion, equilibrium or bottleneck, Neutrality tests i.e. Tajima’s D, Fu’s Fs and Fu and Li’s F and D were carried out to evaluate the demographic effects using DnaSP 6^57–59^. Tajima´s D, Fu´s Fs and Fu and Li’s F and D are the neutrality tests where the significant departure from the null model hypothesis indicates the population expansion or decline. A positive value often indicates decline while the negative value is a sign of population expansion. Further, the mismatch distribution tests identify the trend of variation of observed sequence differences to that expected in the population, which is in equilibrium. The population experiencing recent expansion produces a right-skewed unimodal peak whereas multimodal distributions are indicative of populations at demographic equilibrium. The Harpending’s raggedness index (Rg) and sum of squared deviations (SSD) were calculated to test for demographic expansion under the sudden expansion model using Arlequin version 3.5^60^. The coalescent based Bayesian Skyline Plot (BSP) was reconstructed in BEAST 2 with HKY and empirical base frequencies under the substitution model and the 10^8^ Markov Chain Monte Carlo (MCMC) repetitions. We used a strict molecular clock model to yield effective sample sizes (ESS) taking a substitution rate of 1.2 × 10^–7^ calculated for the red panda. Further to ensure whether or not population structure violated the BSP inferences by misinterpreting the panmixia assumption, we also tested the changes in the effective population size of HRP by re-running BSP after arranging sequences according to two HRP populations i.e. KL-India population (7 haplotypes) and Arunachal Pradesh population (7 haplotypes) following Heller et al.^37^.

## Supplementary Information

Supplementary Information.
