# Supplementary material for: Geological and Pleistocene glaciations explain the demography and disjunct distribution of red panda (A. fulgens) in eastern Himalayas
Source: Sci Rep. 2021 Jan 8;11:65. doi: 10.1038/s41598-020-80586-6 (PMC7794540; doi:10.1038/s41598-020-80586-6)
Supplement: Supplementary file 1 — Supplementary Information. [file 41598_2020_80586_MOESM1_ESM.docx]

**Geological and Pleistocene glaciations explain the demography and disjunct distribution of red panda (*A. fulgens*) in eastern Himalayas**

Supriyo Dalui^1, 2^, Sujeet Kumar Singh^1^, Bheem Dutt Joshi^1^, Avijit Ghosh^1^, Shambadeb Basu^1^, Hiren Khatri^1^, Lalit Kumar Sharma^1^, Kailash Chandra^1^, Mukesh Thakur^1^**^§^**

Supplementary Table S1: Nucleotide variability among different haplotypes observed in the wild collected samples of red panda in the present study from India using mitochondrial control region (mtDNA-CR)

|  | **Nucleotide positions** | | | | | | | | | | | | | | | | | | | | | | | | | | | | | | | | | |
| --- | --- | --- | --- | --- | --- | --- | --- | --- | --- | --- | --- | --- | --- | --- | --- | --- | --- | --- | --- | --- | --- | --- | --- | --- | --- | --- | --- | --- | --- | --- | --- | --- | --- | --- |
| **Haplotype ID** | 15485 | 15493 | 15511 | 15512 | 15518 | 15534 | 15563 | 15564 | 15565 | 15569 | 15572 | 15574 | 15579 | 15582 | 15586 | 15588 | 15621 | 15632 | 15636 | 15644 | 15678 | 15691 | 15695 | 15703 | 15712 | 15723 | 15727 | 15750 | 15752 | 15775 | 15828 | 15887 | 15916 | 15917 |
| complete genome | T | C | C | T | T | C | T | C | A | A | G | T | A | T | C | C | T | G | C | A | C | T | C | C | G | T | C | C | C | A | G | G | A | T |
| Hap_1 | . | . | . | . | C | . | . | T | . | G | A | C | . | C | T | T | . | . | . | . | . | . | . | . | . | . | T | T | . | G | A | . | . | C |
| Hap_2 | . | . | . | . | C | . | . | T | . | G | A | C | . | C | T | . | . | . | T | . | . | . | . | . | . | . | T | T | . | G | A | . | . | C |
| Hap_3 | . | . | . | . | C | . | . | T | . | G | A | C | . | C | T | . | . | . | T | . | T | . | . | . | . | . | T | . | . | G | . | . | . | C |
| Hap_4 | . | . | . | . | C | . | . | T | . | G | A | . | . | . | . | . | C | A | . | . | . | . | . | . | . | . | T | . | . | G | . | . | G | C |
| Hap_5 | . | . | . | . | C | . | . | T | . | G | A | C | . | C | T | . | . | A | . | . | . | . | . | . | . | . | T | T | . | G | A | . | . | C |
| Hap_6 | . | . | . | . | C | . | . | T | . | G | A | . | . | . | . | . | C | A | . | . | . | . | . | . | . | . | . | . | . | . | . | . | G | C |
| Hap_7 | . | . | . | . | C | T | . | T | . | . | A | . | . | C | T | . | . | . | . | . | . | . | . | . | . | . | T | . | . | G | . | . | . | C |
| Hap_8 | . | . | . | . | . | . | . | T | . | G | A | . | . | . | . | . | C | A | . | . | . | A | . | . | . | . | . | . | . | . | . | . | G | C |
| Hap_9 | . | . | . | . | . | . | . | . | . | G | A | . | . | . | T | . | . | A | . | G | . | . | . | T | . | . | . | . | . | . | . | . | . | . |
| Hap_10 | . | . | . | . | C | . | C | T | . | G | . | . | . | . | T | . | . | . | . | . | T | . | T | . | A | . | . | . | . | . | . | . | . | . |
| Hap_11 | . | . | . | . | C | T | . | T | . | . | A | . | . | C | T | . | . | . | . | . | . | . | . | . | . | C | T | . | A | G | . | C | . | C |
| Hap_12 | . | . | . | . | C | T | . | T | . | . | A | . | . | C | T | . | . | . | . | . | . | . | . | . | . | . | T | . | . | G | . | C | . | C |
| Hap_13 | G | G | . | . | C | T | . | T | . | . | A | . | G | C | T | . | . | . | . | . | . | . | . | . | . | . | T | . | . | G | . | C | . | C |
| Hap_14 | G | A | . | G | . | G | . | T | . | . | A | . | . | C | T | . | . | . | . | . | . | . | . | . | . | . | T | . | . | G | . | . | . | C |
| Hap_15 | . | G | . | . | C | . | C | T | G | G | A | . | . | . | T | . | . | . | . | . | T | . | T | . | . | . | . | . | . | . | . | . | . | . |
| Hap_16 | . | . | T | . | C | T | . | T | . | . | A | . | . | C | T | . | . | . | . | . | . | . | . | . | . | . | T | . | . | G | . | . | . | C |
| Hap_17 | . | . | . | . | C | . | . | T | . | . | A | . | . | C | T | . | . | . | . | . | . | . | . | . | . | . | T | . | . | G | . | . | . | C |
| Hap_18 | . | . | . | . | C | . | . | . | . | . | . | . | . | . | T | . | . | . | . | . | . | . | . | . | . | . | . | . | . | G | . | . | . | . |

Supplementary Table S2: Details of sequences downloaded from GenBank for this study

| **Accession No.** | **Location** | **Citations** | **Species** |
| --- | --- | --- | --- |
| AF291586.2 | China | Li et al 2005 | *Ailurus styani* |
| AY849718.1 | China | Li et al 2005 | *Ailurus styani* |
| AY849730.1 | China | Li et al 2005 | *Ailurus styani* |
| AY849727.1 | China | Li et al 2005 | *Ailurus styani* |
| AY849719.1 | China | Li et al 2005 | *Ailurus styani* |
| AY849733.1 | China | Li et al 2005 | *Ailurus styani* |
| AY849731.1 | China | Li et al 2005 | *Ailurus styani* |
| AF291582.2 | China | Li et al 2005 | *Ailurus styani* |
| AY849721.1 | China | Li et al 2005 | *Ailurus styani* |
| AY849716.1 | China | Li et al 2005 | *Ailurus styani* |
| AF291585.2 | China | Li et al 2005 | *Ailurus styani* |
| AF291580.2 | China | Li et al 2005 | *Ailurus styani* |
| AF291579.2 | China | Li et al 2005 | *Ailurus styani* |
| AY849734.1 | China | Li et al 2005 | *Ailurus styani* |
| AY849732.1 | China | Li et al 2005 | *Ailurus styani* |
| AY849715.1 | China | Li et al 2005 | *Ailurus styani* |
| AF291583.2 | China | Li et al 2005 | *Ailurus styani* |
| AY849729.1 | China | Li et al 2005 | *Ailurus styani* |
| AY849725.1 | China | Li et al 2005 | *Ailurus styani* |
| AY849717.1 | China | Li et al 2005 | *Ailurus styani* |
| AF291581.2 | China | Li et al 2005 | *Ailurus styani* |
| AF291584.2 | China | Li et al 2005 | *Ailurus styani* |
| HQ992976.1 | China | Hu et al 2011 | *Ailurus styani* |
| HQ992973.1 | China | Hu et al 2011 | *Ailurus styani* |
| HQ992979.1 | China | Hu et al 2011 | *Ailurus styani* |
| HQ992978.1 | China | Hu et al 2011 | *Ailurus styani* |
| HQ992985.1 | China | Hu et al 2011 | *Ailurus styani* |
| HQ992977.1 | China | Hu et al 2011 | *Ailurus styani* |
| HQ992984.1 | China | Hu et al 2011 | *Ailurus styani* |
| HQ992982.1 | China | Hu et al 2011 | *Ailurus styani* |
| HQ992980.1 | China | Hu et al 2011 | *Ailurus styani* |
| HQ992975.1 | China | Hu et al 2011 | *Ailurus styani* |
| HQ992974.1 | China | Hu et al 2011 | *Ailurus styani* |
| HQ992970.1 | China | Hu et al 2011 | *Ailurus styani* |
| HQ992969.1 | China | Hu et al 2011 | *Ailurus styani* |
| HQ992965.1 | China | Hu et al 2011 | *Ailurus styani* |
| HQ992983.1 | China | Hu et al 2011 | *Ailurus styani* |
| HQ992972.1 | China | Hu et al 2011 | *Ailurus styani* |
| HQ992968.1 | China | Hu et al 2011 | *Ailurus styani* |
| HQ992967.1 | China | Hu et al 2011 | *Ailurus styani* |
| HQ992966.1 | China | Hu et al 2011 | *Ailurus styani* |
| HQ992971.1 | China | Hu et al 2011 | *Ailurus styani* |
| HQ992964.1 | China | Hu et al 2011 | *Ailurus styani* |
| HQ992981.1 | China | Hu et al 2011 | *Ailurus styani* |
| EF517161.1 |  | Cullingham (unpublished) | *Procyon lotor* |
| AB564099.1 |  | Sato et al 2012 | *Procyon cancrivorus* |

Supplementary Table S3. Summary of the Spatial analysis of molecular variance (SAMOVA)

| **K** | **Group** | **FSC** | **FST** | **FCT** | **AG** | **AP (G)** | **WP** |
| --- | --- | --- | --- | --- | --- | --- | --- |
| 2 | SNP, WS, ES, NVNP+TAW, WK, M | 0.234  P=0.00 | 0.545  P=0.01 | 0.405  P=0.03 | 40.56 | 13.90 | 13.90 |
| 3 | SNP, WS+ES, NVNP+TAW, WK, M | 0.081  P= 0.08 | 0.506  P=0 | 0.462  P= 0.007 | 46.23 | 4.38 | 49.40 |
| 4 | SNP, WS+ES+NVNP+ TAW, WK, M | -0.034  P=0.00 | 0.477  P=0.13 | 0.464  P=0 | 49.47 | -1.74 | 52.27 |

SNP=Singhalila NP, WS=West Sikkim, ES= East Sikkim, NVNP=Neora valley NP, TAW= Tawang, WK=West Kameng, M=Menchuka, DB=Dibang valley

Supplementary Figure S1: SAMOVA FCT value reached a plateau at K=3 after which the FCT value increases slowly but single population groups were started to appear and population structure was disappearing. Thus K=3 has been accepted as the final group of population.
